# Diagnostic performance of large language models on the NEJM image challenge: a comparative study with human evaluators and the impact of prompt engineering

**DOI:** 10.3389/fmed.2025.1709413

**Published:** 2026-01-08

**Authors:** Yuchen Zhou, Weiping Wang, Peng Wang, Ke Hu

**Affiliations:** 1 Department of Radiation Oncology, Peking Union Medical College Hospital, Peking Union Medical College, Chinese Academy of Medical Sciences, Beijing, China; 2 Tsinghua Medicine, School of Medicine, Tsinghua University, Beijing, China

**Keywords:** artificial intelligence in medicine, clinical decision support, medical education, multimodal large language models, NEJM image challenge

## Abstract

**Introduction:**

Multimodal large language models (LLMs) that can interpret clinical text and images are emerging as potential decision-support tools, yet their accuracy on standardized cases and how it compares with human performance across different difficulty levels remains largely unclear. This study aimed to rigorously evaluate the performance of four leading LLMs on the 200-item New England Journal of Medicine (NEJM) Image Challenge.

**Methods:**

We assessed OpenAI o4-mini-high, Claude 4 Opus, Gemini 2.5 Pro, and Qwen 3, and benchmarked the top model against three medical students (Years 5–7) and an internal-medicine attending physician under identical test conditions. Additionally, we characterized the dominant error types for OpenAI o4-mini-high and tested prompt engineering strategies for potential correction.

**Results:**

Our results suggest that OpenAI o4-mini-high achieved the highest overall accuracy of 94%. Its performance remained consistently high across easy, moderate, and difficult cases. The human accuracies in this cohort ranged from 38.5% for three medical students to 70.5% for an attending physician—all significantly lower than OpenAI o4-mini-high. An analysis of OpenAI o4-mini-high’s 12 errors revealed that most (83.3%) were outputs reflecting lapses in diagnostic logic rather than input processing. Notably, simple prompting techniques like chain-of-thought and few-shot learning corrected over half of these initial errors.

**Conclusion:**

Within the context of this standardized challenge, a leading multimodal LLM delivered high diagnostic accuracy that surpassed the scores of both peer models and the recruited human participants. However, these results should be interpreted as evidence of pattern recognition capabilities rather than human-like clinical understanding. While further validation on real-world data is warranted, these findings support the potential utility of LLMs in educational and standardized settings, highlighting that most residual errors are due to logic gaps that can be partly mitigated by refined user prompting, emphasizing the importance of human-AI interaction for maximizing reliability.

## 1 Introduction

Large language models (LLMs) have rapidly reshaped natural language processing and are increasingly applied in medicine, where they can support tasks from knowledge retrieval to clinical question answering (1). Leveraging scale and multi-stage training and alignment techniques, modern general-purpose LLMs such as GPT-4 achieve human-level or near-human-level performance on a range of professional medical benchmarks (2).

Building on these foundations, domain-specialized medical variants (e.g., Med-PaLM and Med-PaLM 2) have exceeded the passing thresholds on the United States Medical Licensing Examination (USMLE) style questions and narrowed gaps with clinician answers through targeted prompting and refinement (3). Other competitive models such as Claude and Gemini (including its medical adaptation Med-Gemini (4)) have also showed strong performance in tasks requiring reasoning-like processing. Recent research indicates that these models show generally high but variable performance across different clinical scenarios, including stroke care (5).

In parallel, regionally developed LLMs, including DeepSeek-R1, ERNIE Bot, and Qwen, have begun to demonstrate comparable strengths, particularly in language-context-matched settings (e.g., Chinese medical licensing examinations, CNMLE). This finding suggests that training data composition and linguistic alignment could potentially influence real-world diagnostic accuracy (6, 7).

Because medical licensing and certification examinations (e.g., the USMLE, CNMLE) are structured, high-stakes assessments that test both basic knowledge and clinical reasoning, researchers have selected them as primary benchmarks for evaluating clinical knowledge and decision-making capacity of LLMs. However, several important limitations of these benchmarks need to be addressed. First, most prior studies of medical LLMs have focused on text-only question formats (e.g., USMLE-style multiple-choice items), which do not capture the full spectrum of diagnostic output generation required in real-world practice, particularly scenarios that combine multimodal inputs (8). Second, because most LLMs’ training corpora remain undisclosed, they may include original or near-duplicate versions of standardised benchmark exam questions (e.g., USMLE) (9). As a result, observed high performance could result from memorization rather than true generalization.

Unlike conventional text-only question sets, the New England Journal of Medicine (NEJM) Image Challenge presents an interactive, widely used quiz format that pairs high-quality clinical images with concise patient vignettes. By regularly updating its case library, it minimizes the chance of overlap with opaque training data. The platform also delivers immediate, expert-annotated feedback including the correct answer, a focused explanation, and links to relevant literature, which establishes a clear reference standard for diagnostic performance. For these reasons, the NEJM Image Challenge is recognized as a reliable resource in medical education and self-assessment (10).

Researchers have adopted the NEJM Image Challenge as a practical benchmark for evaluating the diagnostic and multimodal reasoning capabilities of LLMs. Early work assessing GPT-4-based ChatGPT on the Image Challenge showed high case-level accuracy with image-plus-vignette inputs (10). Subsequent multimodal evaluations expanded this comparison to more recent vision-enabled models. Studies of GPT-4 V and other contemporary models (e.g., Gemini Pro, Claude 3 family) demonstrated that vision-augmented LLMs can rival or exceed average human performance on Image Challenge cases, although collective human judgment still often outperformed individual models (11, 12). This comparative landscape has been further broadened by domain-specific benchmarks, such as in gastroenterology, which have rigorously evaluated a diverse array of proprietary and open-source models (including Llama, Mistral, and quantized variants) (13). However, high performance metrics warrant cautious interpretation; recent critical analyses have exposed “hidden flaws” behind the expert-level accuracy of multimodal models, revealing that correct diagnoses may sometimes stem from superficial associations rather than robust clinical reasoning (14). Consistent with these findings, comparative work in radiology contexts also found that LLMs could outperform medical students on NEJM Image Challenge cases, reflecting the promise of these LLMs and also the need for domain-specific validation (15). A summary of key prior studies evaluating vision-augmented LLMs in medical contexts, including their methodologies and main findings, is provided in Supplementary Table S1.

Despite encouraging prior results, key gaps remain. Earlier work has often used limited subsets of NEJM Image Challenge cases or only a few models and does not fully describe how modern multimodal LLMs perform under different input conditions, failing to capture the rapid evolution of multimodal capabilities. More importantly, it is necessary to acknowledge the distinction between standardized benchmarks and clinical reality. The NEJM Image Challenge consists of high-quality images that do not fully represent the size, variation, and image quality (e.g., noise, artifacts) of routine real-world medical imaging. However, this standardized nature provides an essential foundation for validation. Demonstrating robust diagnostic reasoning on high-quality cases is a critical prerequisite before deploying models to handle the heterogeneity and noise of raw clinical data. To address these gaps, this study compares four current multimodal LLMs—OpenAI o4-mini-high, Claude 4 Opus, Gemini 2.5 Pro, and Qwen 3—using a broad NEJM Image Challenge set (200 cases). The analysis assesses each model’s diagnostic accuracy with image-only, text-only, and combined inputs, aiming to provide a comprehensive assessment of their multimodal capabilities of processing clinical information, which serves as a stepping stone toward future domain-specific validation.

## 2 Materials and methods

### 2.1 Study resources

The question bank was derived from the *NEJM Image Challenge*, a publicly accessible, weekly updated series of clinical cases that pairs diagnostic images with brief patient vignettes and a single prompt. A total of 200 cases were retrieved from the *NEJM Image Challenge* website, covering the period from August 5, 2021, to May 29, 2025. All included cases in this study contained both images and corresponding clinical vignette. Images were retrieved from the source in their original format (JPEG). To evaluate the models, these files were manually uploaded via the standard image attachment interface of each platform. A descriptive analysis of the dataset revealed that the input images had a fixed width of 1,274 pixels and a variable height with a mean of 920.4 ± 417.8 pixels. The average file size was 222.9 ± 102.6 KB, representing typical high-quality clinical photographs and radiographic scans. The data used was public and de-identified. The usage was strictly for non-commercial research evaluation, aligning with standards set by previous studies in the domain (10, 11). To comply with copyright regulations, no original images from these cases are reproduced in this manuscript. Instead, specific cases are referenced by their publication dates to allow verification on the official website. To aid reader understanding of the data structure, a schematic illustration of the task format is provided in Supplementary Figure S1.

### 2.2 Stratification by case difficulty

To evaluate model performance across varying levels of difficulty, we retrieved historical human participant accuracy data for each question from the official NEJM Image Challenge website. Based on these performance metrics, the 200-item dataset was stratified into three difficulty tiers: “Easy” cases (*n* = 67; participant accuracy 55–83%), “Moderate” cases (*n* = 67; accuracy 45–55%), and “Difficult” cases (*n* = 66; accuracy 31–45%).

### 2.3 LLMs

This study evaluated four contemporary LLMs: OpenAI o4-mini-high (OpenAI, United States), Claude 4 Opus (Anthropic, United States), Gemini 2.5 Pro (Google/DeepMind, United States & United Kingdom), and Qwen 3 (Alibaba Cloud, China). Each model was accessed via its official web-based chat interfaces, not via APIs. No additional task-specific fine-tuning was applied. Data were collected between June 1 and July 1, 2025. We utilized the platforms’ default sampling parameters (e.g., temperature and top-p) as enforced by the respective vendors during the data collection period. Specific numerical values for these parameters are undisclosed for web-based interfaces. All models received the same standardized prompt format (see “Prompt design”) consisting of the NEJM Image Challenge clinical vignette and associated image input. Key public specifications of the four LLMs evaluated in this study are summarized in Table 1.

**Table 1:** Key public information of the LLMs evaluated in this study.

Model	Developer/Release (2025)	Context window (tokens)	Reported parameters	Key source
OpenAI o4-mini-high	OpenAI, Apr 16	≈ 200,000	Not disclosed	Introducing-o3-and-o4-mini 2025 (16)
Claude 4 Opus	Anthropic, May 22	200,000	Not disclosed	Models overview 2025 (17)
Gemini 2.5 Pro	Google/DeepMind, Mar 25	1,000,000	Not disclosed	Gemini 2.5: Our most intelligent AI model 2025 (18)
Qwen 3 (235B-A22B)	Alibaba Cloud, Apr 29	32,768 (native)	235 B (22 B active)	Qwen3-235B-A22B 2025 (19)

### 2.4 Human comparator group

To establish a comparative benchmark, a cohort of four human evaluators was recruited via voluntary participation to complete the same 200-item question bank. The cohort comprised three medical students enrolled in an 8-year MD program in China, representing the 5th, 6th, and 7th academic years, respectively. All student participants had completed the comprehensive theoretical curriculum in basic and clinical sciences and were currently engaged in clinical rotations. The fourth participant was an attending physician with an MD degree who had completed 3 years of standardized residency training in internal medicine. All participants evaluated the cases independently, utilizing the same clinical vignettes and images as the models, without access to external references or time constraints.

### 2.5 Prompt design

To assess the impact of different input modalities, we employed a zero-shot persona-based Prompting strategy. Each model was tested under three conditions: combined image + text, text-only, and image-only. The prompts were designed to establish a specific expert persona (“Act as an experienced physician and professor”), thereby aligning the models’ outputs with professional diagnostic output standards. No additional context or few-shot examples were provided. To ensure reproducibility, the exact prompts used for each condition are listed below:

Prompt 1 (combined): “Act as an experienced physician and professor at a prestigious university hospital. Your task is to answer questions based on medical images and patient case descriptions. Utilize your expertise to accurately interpret the information and provide the most likely diagnosis or answer choice.” Input: Clinical vignette + corresponding images.

Prompt 2 (text only): “Act as an experienced physician and professor at a prestigious university hospital. Your task is to answer questions based on patient case descriptions. Utilize your expertise to accurately interpret the information and provide the most likely diagnosis or answer choice.” Input: Clinical vignette only.

Prompt 3 (image only): “Act as an experienced physician and professor at a prestigious university hospital. Your task is to answer questions based on medical images. Utilize your expertise to accurately interpret the information and provide the most likely diagnosis or answer choice.” Input: Corresponding images only.

### 2.6 Prompt engineering strategy

To enhance response accuracy and standardize model outputs, we implemented a structured prompt engineering approach combining Chain-of-Thought (CoT) protocols and few-shot demonstrations.

Chain-of-Thought (CoT) Design: The prompt explicitly instructed the model to adhere to a five-stage clinical reasoning framework: (1) Observation; (2) Hypothesis generation; (3) Evidence weighing; (4) Differential narrowing; and (5) Conclusion. The model was required to label each stage sequentially and provide a final answer only after completing this full reasoning process. To enforce this structure, we utilized a natural language formatting instruction within the prompt. The prompt provided a specific output template, guiding the model to generate a standardized, step-by-step textual response.

Few-Shot Prompting: To further guide model performance via in-context learning, we provided three representative questions from the NEJM Image Challenge (published on May 29, May 22, and May 15, 2025) as few-shot exemplars. For these examples, the model was presented with the full case details along with the corresponding correct answers and official explanations.

The complete text of the CoT prompts and the specific few-shot examples used are detailed in Supplementary Material S1.

### 2.7 Qualitative error analysis

To investigate the underlying mechanisms of diagnostic failure, a qualitative analysis was performed on the subset of questions answered incorrectly by the OpenAI o4-mini-high model. We have examined the model’s generated reasoning chains and categorized the errors into two primary domains:

1. Input Processing/Multimodal Alignment: This category included errors stemming from a failure to accurately process the visual input, the textual vignette, or the association between the two. It included instances of visual hallucination, missed key visual features, or misalignment where diagnoses were based on incomplete or mismatched multimodal information.
2. Diagnostic Logic/Decision-making Output: This category included errors where the input data appeared to be correctly identified, but the subsequent logic progression was flawed. Specific examples included assigning disproportionate weight to minor findings, premature closure on common diagnoses, failure to exclude relevant differentials, or logical fallacies in the deduction process despite the apparent retrieval of relevant clinical concepts.

### 2.8 Statistical analysis

Model performance was compared using chi-square tests with Bonferroni correction for multiple comparisons and all reported *p*-values were adjusted accordingly. 95% confidence intervals were calculated using the Wilson score method. Statistical analyses were performed in SPSS version 30.0.0. Figures were generated using GraphPad Prism version 10.4.0.

## 3 Results

### 3.1 OpenAI o4-mini-high achieved the highest overall accuracy on NEJM image challenge questions across input modalities

To assess the specific contributions of visual versus textual information and identify potential text-dominance, we evaluated performance under three input conditions: combined patient vignette and image (“text + image”), vignette only (“text only”), and image only. This comparative analysis isolates the diagnostic value added by each modality, allowing for a statistical assessment of whether the models effectively leverage visual data or rely primarily on the clinical vignette.

**Figure 1:**
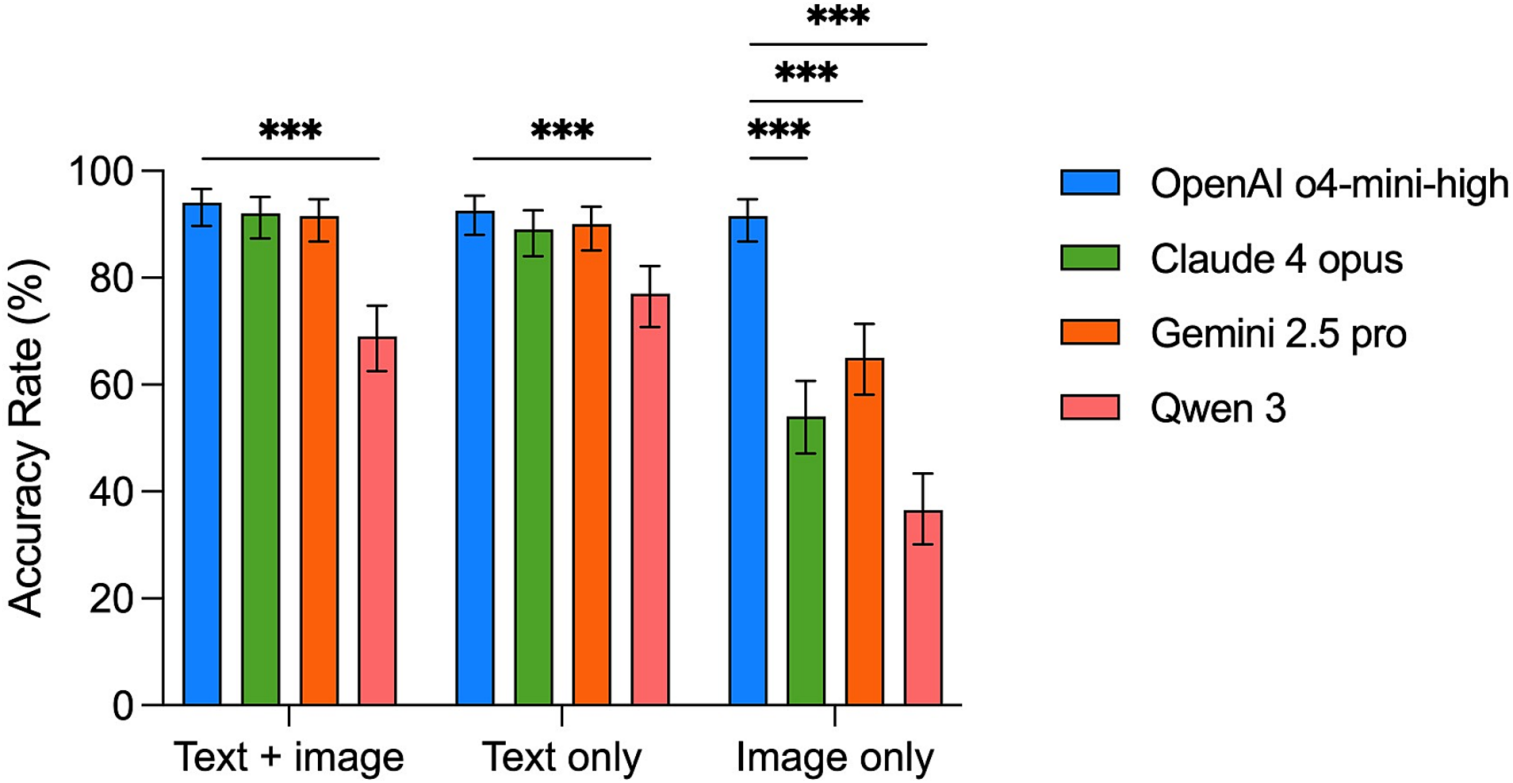
Overall accuracy of four LLMs on NEJM Image Challenge questions under three input conditions. Bars show case-level accuracy (%) for each model with text + image, text-only, or image-only inputs and corresponding prompts. Error bars represent 95% confidence intervals calculated using the Wilson score method. Chi-square test with Bonferroni correction, *** adjusted *p* < 0.001.

As shown in Figure 1 and Table 2, all models achieved their highest accuracy with the combined input (text + image). Under this condition, OpenAI o4-mini-high (94%, 188/200) demonstrated performance comparable to Claude 4 Opus (92%, 184/200, *p* = 0.43) and Gemini 2.5 Pro (91.5%, 183/200, *p* = 0.34). In contrast, Qwen 3 reached 69% (138/200), which was significantly lower than the other models (*p* < 0.001).

**Table 2:** Overall accuracy of four LLMs.

Model	Input condition	Accuracy (%)	95% CI (%)	Adjusted *p*
OpenAI o4-mini-high	Text + image	94	89.7–96.6	Reference
	Text only	92.5	88.0–97.0	1.00
	Image only	91.5	86.8–96.2	0.88
Claude 4 Opus	Text + image	92	87.4–96.6	Reference
	Text only	89	84.0–93.6	0.79
	Image only	54	47.3–60.7	<0.001
Gemini 2.5 Pro	Text + image	91.5	86.8–96.2	Reference
	Text only	90	84.7–95.3	1.00
	Image only	65	58.1–71.4	<0.001
Qwen 3	Text + image	69	62.5–75.5	Reference
	Text only	77	70.8–83.2	0.18
	Image only	36.5	30.1–42.9	<0.001

Adjusted *p*-values are from chi-square tests with Bonferroni correction, comparing each input condition (text-only or image-only) to the corresponding model’s performance with text + image input.

When evaluating the contribution of textual information, comparisons between the text-only and combined conditions revealed distinct behavioral patterns. For OpenAI o4-mini-high, Gemini 2.5 Pro, and Claude 4 Opus, removing visual data resulted in minor, non-significant performance declines of 1.5, 1.5, and 3.0%, respectively (OpenAI and Gemini: *p* = 1.00, Claude: *p* = 0.79; Table 2). Conversely, Qwen 3 exhibited a paradoxical improvement in the text-only condition, rising to 77.0% (154/200) compared to 69% in the combined setting (*p* = 0.07), although it remained significantly lower than the top-performing models (*p* < 0.001, Figure 1).

In the image-only situation, Claude 4 Opus (54.0%), Gemini 2.5 Pro (65.0%), and Qwen 3 (36.5%) all showed statistically significant performance reduction compared to their multimodal baselines (*p* < 0.001 for all, Table 2). In contrast, OpenAI o4-mini-high maintained high performance (183/200, 91.5%) even without textual descriptions, whose accuracy was significantly higher than the other three models (*p* < 0.001, Figure 1).

### 3.2 OpenAI o4-mini-high demonstrated higher accuracy rates over the other models in the most challenging cases

In addition to the correct answers and reference knowledge point, participant accuracy data for each question are also publicly available on the NEJM Image Challenge official website. As defined in the Material and methods, subsection “Stratification by case difficulty”, performance was analysed across easy, moderate, and difficult tiers. The accuracy of the four models was then assessed across these difficulty tiers using the combined text + image input, as this setup best reflects real-world clinical scenarios.

**Figure 2:**
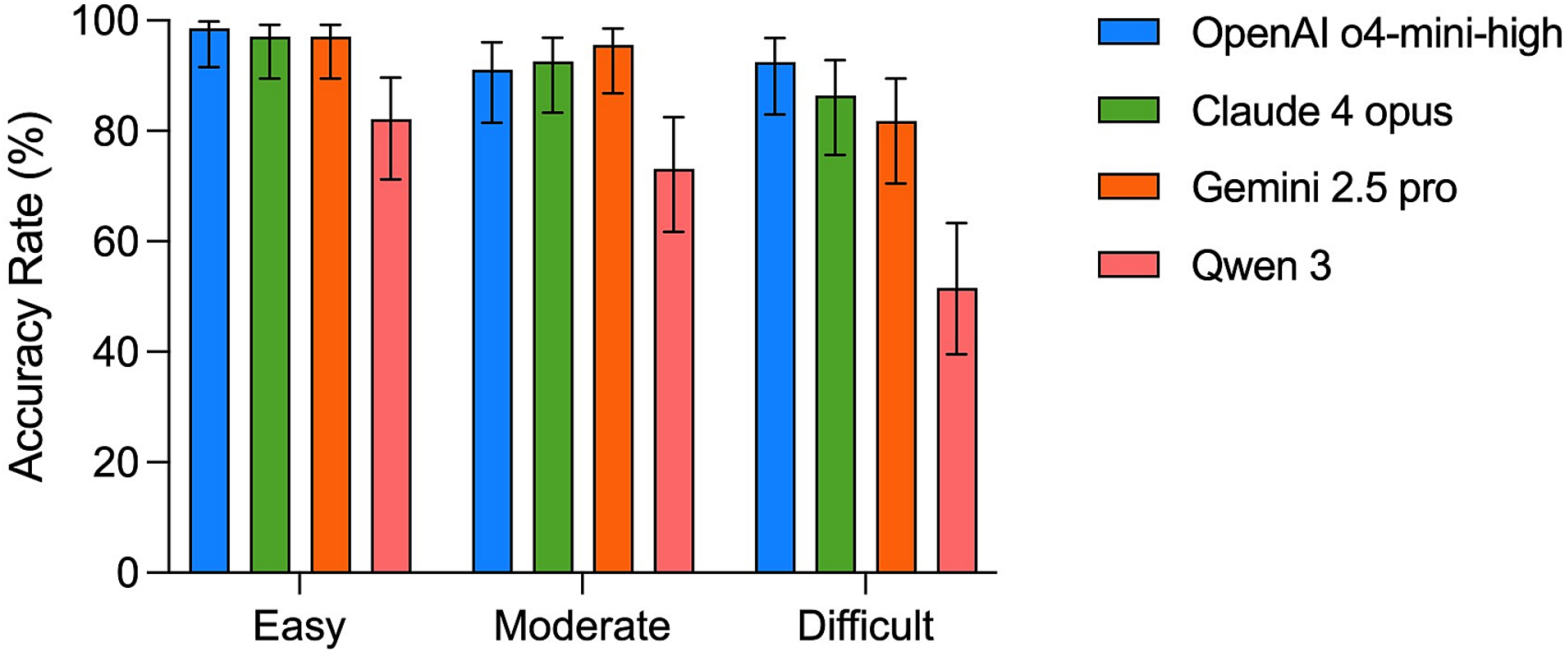
Accuracy of four LLMs under combined text + image input, stratified by question difficulty. Bars represent the percentage of correctly answered cases for the four models across easy (*n* = 67), moderate (*n* = 67), and difficult questions (*n* = 66). Error bars indicate 95% confidence intervals.

In the easy tier (*n* = 67), OpenAI o4-mini-high achieved 98.5% accuracy (66/67, 95% CI: 91.6–99.8%), Claude 4 Opus 97.0% (65/67, 89.4–99.2%), and Gemini 2.5 Pro 97.0% (65/67, 89.4–99.2%, Figure 2). Qwen 3 scored 82.1% (55/67, 71.2–89.6%), indicating a notable gap on straightforward cases.

In the moderate tier (*n* = 67), accuracy declined for all models, though the top three models remained closely clustered. OpenAI o4-mini-high scored 91.0% (61/67, 81.5–96.1%), Claude 4 Opus 92.5% (62/67, 83.3–96.9%), and Gemini 2.5 Pro 95.5% (64/67, 86.8–98.6%), while Qwen 3 fell to 73.1% (49/67, 61.7–82.4%, Figure 2).

In the difficult tier (*n* = 66), performance diverged further. OpenAI o4-mini-high remained consistent at 92.4% (61/66, 83.0–96.8%), whereas Claude 4 Opus and Gemini 2.5 Pro dropped to 86.4% (57/66, 75.6–92.8%) and 81.8% (54/66, 70.4–89.4%), respectively. The accuracy of Qwen 3 fell to 51.5% (34/66, 39.6–63.3%, Figure 2), which underscores its reduced performance on the most challenging image-based cases. Notably, in this tier of cases, OpenAI o4-mini-high demonstrated higher accuracy scores over the other models (vs. Claude 4 opus: +6%, *p* = 0.40; vs. Gemini 2.5 pro: +10.6%, *p* = 0.12; vs. Qwen 3: +40.9%, *p* < 0.001, Figure 2) when compared to other tiers of cases.

In summary, among the four models, OpenAI o4-mini-high maintained stable accuracy even in the difficult tier, whereas the other three models showed a general decline in accuracy as case difficulty increased.

### 3.3 OpenAI o4-mini-high showed consistently high accuracy across medical specialties

To further explore performance patterns of the four models, the 200 cases were classified into 21 medical specialties based on their clinical focus (Supplementary Table S2). Dermatology (15%, 30/200) and infectious diseases (14.5%, 29/200) represented the largest proportions, followed by pediatrics (9.5%, 19/200) and clinical immunology (8.5%, 17/200). For subsequent analyses, we focused on specialties with a relatively higher proportion (≥10% of the question bank) to ensure stable estimates of accuracy. The performance of the four models was then compared within these medical specialties.

**Figure 3:**
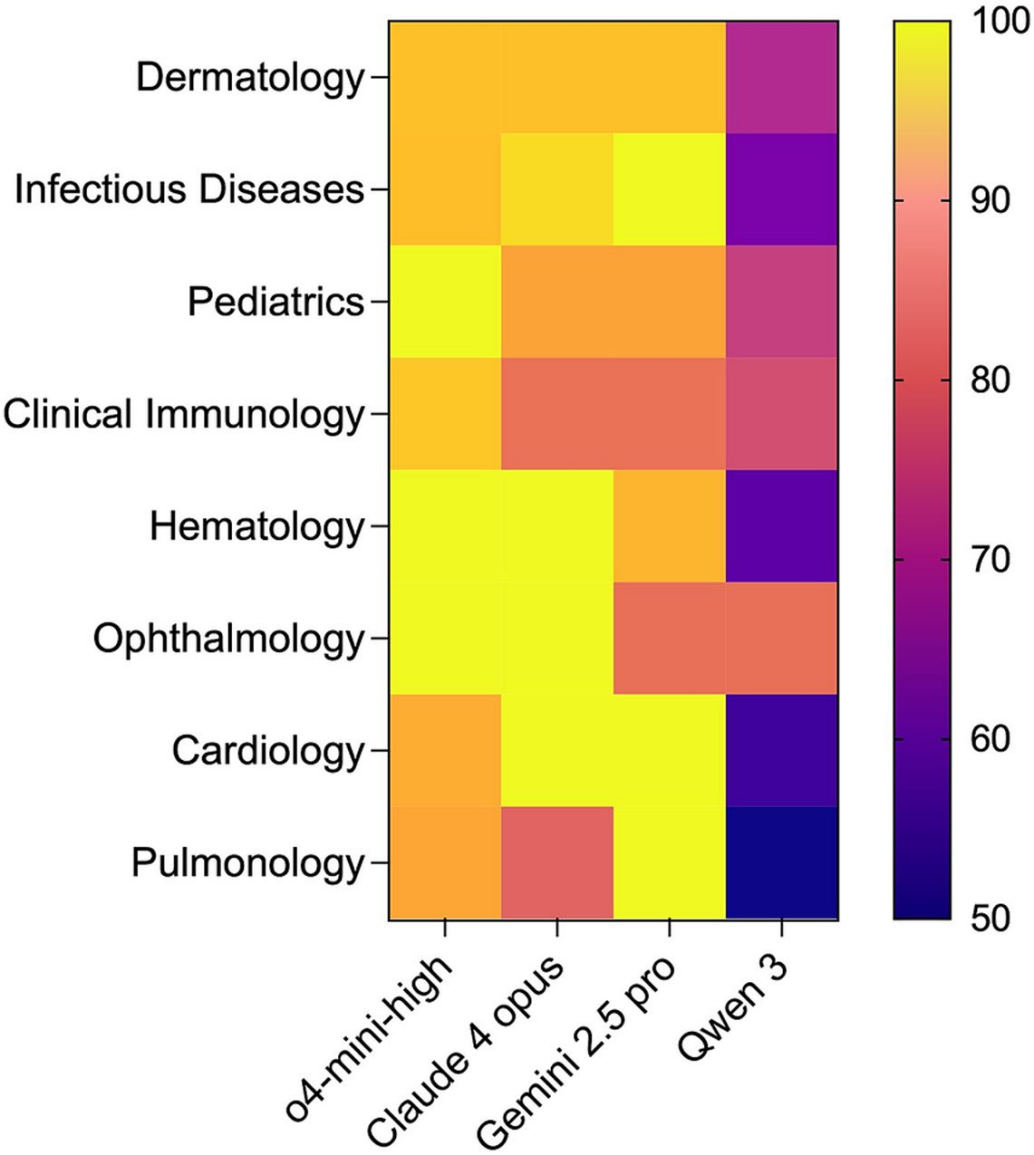
Heatmap of accuracy for four LLMs across different medical specialties. The x-axis lists the four evaluated models (OpenAI o4-mini-high, Claude 4 Opus, Gemini 2.5 Pro, and Qwen 3), and the y-axis shows case subgroups categorized by the medical specialty. Color intensity reflects the percentage accuracy for each model-specialty combination, with warmer colors indicating higher accuracy.

As shown in Figure 3, OpenAI o4-mini-high consistently achieved high accuracy across medical specialties, ranging from 90.9% in cardiology (10/11) to 100% in pediatrics (19/19), 100% in hematology (12/12), and 100% in ophthalmology (11/11). Claude 4 Opus also showed strong performance (80.0–100%), with the highest accuracy in hematology (100%, 12/12), ophthalmology (100%, 11/11), and cardiology (100%, 11/11), but lower accuracy in pulmonology (80.0%, 8/10) and clinical immunology (14/17, 82.4%). Gemini 2.5 Pro maintained high performance in most specialties, including 100% in infectious diseases (29/29), 100% in cardiology (11/11), and 100% in pulmonology (10/10), but scored lower in ophthalmology (81.8%, 9/11) and hematology (91.7%, 11/12). Qwen 3 displayed more variability compared to other models, achieving moderate accuracy in ophthalmology (81.8%, 9/11) and pediatrics (73.7%, 14/19), but lower scores in dermatology (70.0%, 21/30), infectious diseases (62.1%, 18/29), hematology (58.3%, 7/12), cardiology (54.6%, 6/11), and pulmonology (50%, 5/10).

### 3.4 OpenAI o4-mini-high achieved superior diagnostic accuracy when compared to human participants in this study

To enable a preliminary benchmark comparison beyond model-to-model performance, we also included human participants in our study. Specifically, we assessed how the model performed relative to medical students and a practicing physician when answering the same question bank. Full details regarding participant recruitment and testing conditions are provided in the Material and methods section, subsection “Human comparator group”.

**Figure 4:**
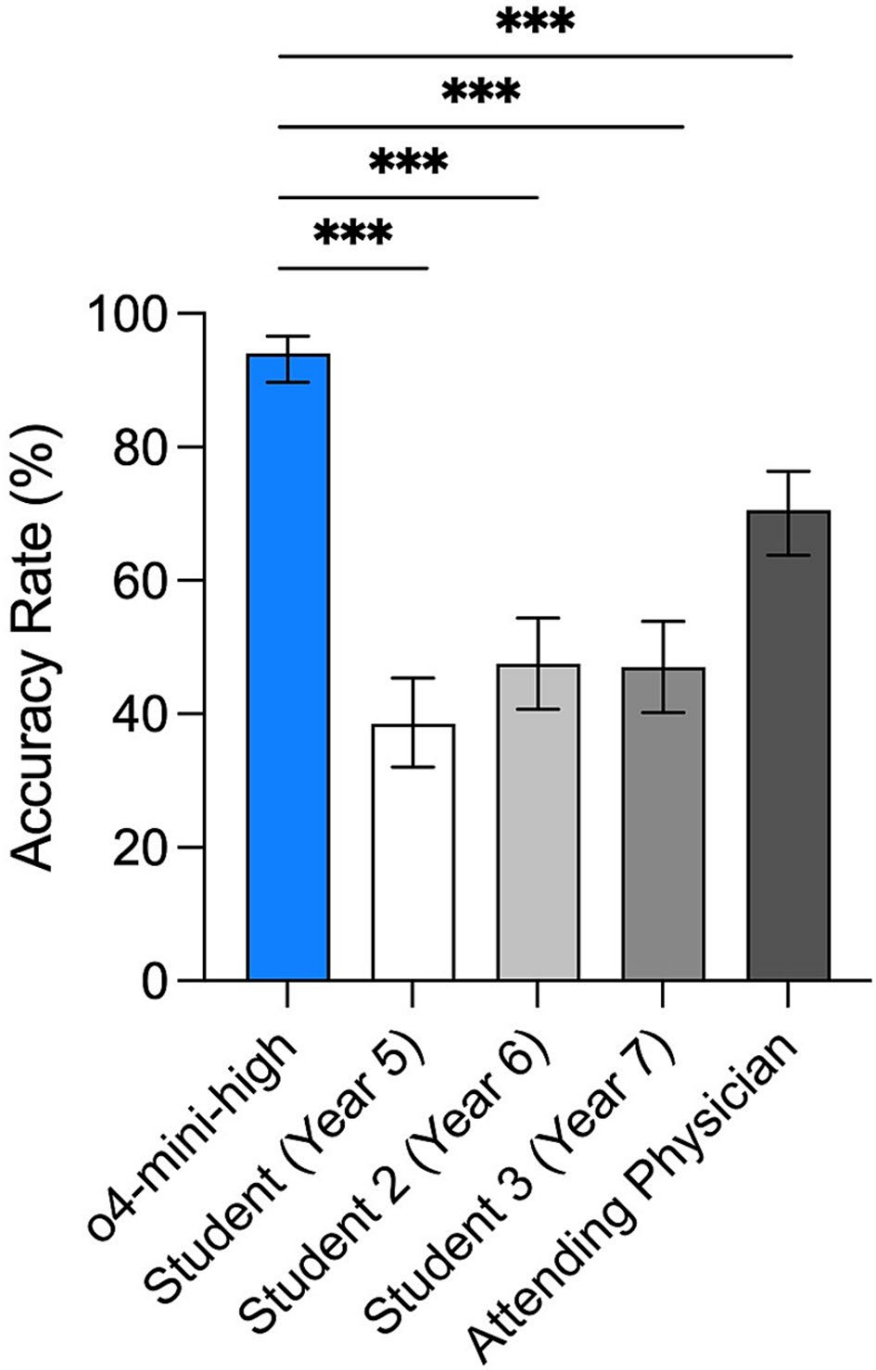
Comparison of accuracy rates between OpenAI o4-mini-high and medical trainees of different levels. Bar plot showing the accuracy rates with 95% CI of OpenAI o4-mini-high compared with three medical students at different training stages (Year 5 to Year 7) and one attending physician. Error bars represent 95% confidence intervals calculated using the Wilson score method. Chi-square test with Bonferroni correction, ***adjusted *p* < 0.001.

To evaluate diagnostic performance, we compared the accuracy of OpenAI’s o4-mini-high model with these four participants. As previously described, the o4-mini-high model correctly answered 188 out of 200 questions, yielding an accuracy of 94%. In contrast, the Year 5 medical student answered 77 questions correctly (38.5, 95% CI: 32.0–45.4%), the Year 6 student got 94 questions correct (47, 95% CI: 40.2–53.9%), and the Year 7 student answered 95 correctly (47.5, 95% CI: 40.7–54.4%). The attending physician achieved a 70.5% accuracy with a 95% CI of 63.8–76.4% (141/200, Figure 4). Overall, the o4-mini-high model achieved significantly higher scores than human participants in this cohort (*p* < 0.001 for all pairwise comparisons). While the sample size precludes broad generalization, these results suggest that the model’s accuracy on this specific dataset exceeded that of the sampled trainees and physician.

### 3.5 Prompt engineering improved accuracy on the 12 misanswered cases with OpenAI o4-mini-high

Among the 200 cases, OpenAI o4-mini-high made 12 errors when given text + image inputs. To further analyse the nature of these errors, we reviewed the model’s responses for the 12 questions and grouped them into two main categories: “Input processing/Multimodal alignment” and “Diagnostic logic/Decision-making output”. The details of these classifications were provided in the Material and methods section, subsection “Prompt engineering strategy”.

For OpenAI o4-mini-high, 10 questions (83.3%) were answered incorrectly due to errors in output generation related to diagnostic logic/decision-making, while 2 questions (16.7%) were due to input processing/multimodal alignment issues (Table 3). Detailed responses and the classification of error types for these questions are provided in Supplementary Table S3.

**Table 3:** Error type analysis of 12 misanswered cases by OpenAI o4-mini-high.

Error type	Number of cases	% of Errors	Description
Clinical reasoning/decision-making error	10	83.3%	Incorrect weighting of clues, overreliance on common diagnoses, or failure to exclude alternatives
Input comprehension/multimodal alignment	2	16.7%	Misinterpretation of question text, image, or their integration

Building on this analysis, while most errors were classified as deficiency in output logic, we then tested whether accuracy could be improved with prompt engineering strategies designed to structure the output generation process, including CoT and few-shot prompting. CoT prompting is a technique that encourages a language model to generate a logical sequence of steps. By guiding the model to describe its observations, hypotheses, evidence evaluation, and reasoning, CoT prompts have been shown to improve accuracy, especially for complex tasks such as medical question answering (3, 20).

In our experiments, we first designed a structured CoT prompt to guide OpenAI o4-mini-high in processing multimodal medical questions. Of the 12 questions that the model initially answered incorrectly, six were correctly answered when the CoT prompt was applied (Table 4). The effect of few-shot prompting was also examined. Few-shot prompting is a technique in which a language model is provided with a small number of input–output examples as part of the prompt to guide its responses on similar tasks. Under the few-shot prompting condition, the model correctly answered 7 of these 12 questions (Table 4). Detailed outputs for these 12 questions, along with the full content of the prompts used, are provided in Supplementary Material S1.

**Table 4:** Impact of CoT and few-shot prompt engineering strategies on OpenAI o4-mini-high (*n* = 12).

Prompt strategy	Corrected cases	Notes
Chain-of-thought (CoT) prompting	6/12	Structured stepwise reasoning (Observation → Hypothesis → Evidence → Narrowing → Conclusion)
Few-shot prompting	7/12	Provided 3 example Q&A pairs with explanations from NEJM Image Challenge

## 4 Discussion

In this study, we conducted a comprehensive evaluation of four multimodal LLMs on 200 clinical cases from the NEJM Image Challenge. Our findings suggest that the contemporary models, particularly OpenAI o4-mini-high, achieved accuracy scores that surpassed those of human participants on this question bank. Furthermore, our analysis across case difficulties, and medical specialties provides insight into their performance patterns on standardized clinical tasks.

Our primary finding is the performance of OpenAI o4-mini-high, which achieved 94% accuracy with combined image and text inputs (Figure 1). This finding builds upon previous studies that showed high performance of earlier models like GPT-4 on the NEJM Image Challenge (10, 11). A key contribution of this study is the detailed analysis of how different input modalities could potentially affect model performance. Comparisons between combined and text-only inputs revealed that for Claude 4 Opus and Gemini 2.5 Pro, removing visual data resulted in no statistically significant performance decline (*p* > 0.05, Table 2). This finding indicates that their diagnostic outputs are predominantly driven by textual processing, where visual data provides minimal additive value to the clinical vignette. This aligns with the known strength of LLMs in natural language processing but points to a limitation in effectively leveraging visual information for these models. In contrast, OpenAI o4-mini-high maintained comparable accuracy of 91.5% with image-only inputs, scoring significantly higher than peer models in the visual-only condition (Figure 1). While this may suggest effective visual pattern matching, it is important to interpret this result with caution. High accuracy on images without clinical vignettes may also indicate potential training data contamination or memorization, as real-world diagnosis rarely relies solely on visual information.

Interestingly, Qwen 3 exhibited a lower performance in the multimodal setting compared to the text-only baseline. While determining the precise cause is beyond the scope of this study, we hypothesize that this may be attributed to visual hallucinations (21, 22). In scenarios where the clinical vignette provides strong diagnostic cues, visual inputs that are not perfectly aligned or interpreted may introduce noise or conflicting signals, reducing the accuracy of the final output. This underscores the challenge of effective cross-modal alignment in emerging vision-augmented LLMs.

Building on overall accuracy findings, Figure 2 further breaks down performance by case difficulty. As case complexity rises from easy to moderate to difficult, Claude 4 Opus, Gemini 2.5 Pro, and Qwen 3 all showed drops in accuracy, whereas OpenAI o4-mini-high remained stable (Figure 2). This stability suggests that o4-mini-high maintains output consistency even under challenging multimodal settings. Complementing that, Figure 3 presents a specialty-level heatmap across the 21 clinical domains. o4-mini-high achieved over 90% accuracy in every high-frequency specialty, while the other three models exhibited greater variability (Figure 3). Together, these results underscore o4-mini-high’s consistent accuracy scores across medical domains and highlight its potential utility across diverse clinical areas within the context of standardized benchmarks.

In the head-to-head evaluation in Figure 4, o4-mini-high achieved an accuracy of 94%, scoring higher than both the medical students (38.5–47.5%) and the internal medicine attending physician (70.5%) recruited for this study. These findings indicate that, within the constraints of this specific cohort and standardized image-based question set, the model demonstrated accuracy scores exceeding that of an experienced clinician. Rather than replacing human judgment, however, such a tool could function as an adjunct in educational settings, offering real-time feedback to trainees and helping to mitigate variability in diagnostic performance across different levels of training.

Furthermore, the potential for inherent biases in the models’ training data, which may stem from the different origins of their developers, warrants discussion. In our study, three of the models (OpenAI o4-mini-high, Claude 4 Opus, and Gemini 2.5 Pro) were developed by US-based companies, while Qwen 3 was developed by companies in China. Previous research shows that regionally developed models often have a “home-field advantage”. For example, Chinese-developed LLMs like Qwen and Baichuan have demonstrated superior performance on local benchmarks, such as the Chinese National Nursing and Medical Licensing Examinations (23, 24). Conversely, the significant performance gap observed for Qwen 3 in this study might be partially explained by a similar bias. The NEJM Image Challenge is an English-language benchmark based on western medical context. It is possible that Qwen 3’s training data have less exposure to the specific terminologies, case presentations, and diagnostic patterns in this dataset, when compared to US-developed models. This highlights a critical point for the global deployment of medical AI. A model’s accuracy in specific benchmarks may be highly dependent on the alignment between its training corpus and the specific clinical, cultural, and linguistic context.

Finally, our error analysis provided additional insight into the top-performing model in this question bank. The majority of OpenAI o4-mini-high’s errors were classified as outputs reflecting lapses in diagnostic logic (10/12, Table 3) rather than fundamental input processing issues. Encouragingly, these performance gaps could be partially addressed. Advanced prompting strategies like CoT and few-shot learning corrected over half of these initial errors (Table 4). This outcome is consistent with findings from other studies demonstrating that structured output prompts and targeted refinement can significantly enhance LLM accuracy in complex medical question answering, narrowing the gap with clinician-level performance (3, 20).

Despite these promising results, it is important to acknowledge several limitations of our study. First, the potential for training data contamination remains a significant challenge in evaluating LLMs. Given that NEJM Image Challenge cases are widely circulated online, it is possible that older cases were included in the models’ training corpora. Although we prioritized the most recent cases available (up to May 2025) to mitigate this issue, the risk of prior exposure cannot be entirely ruled out. Therefore, the models’ performance should be interpreted as a combination of pattern recognition capabilities and potential knowledge retrieval.

Second, standardized quiz accuracy does not directly translate to real-world clinical decision-making. While the NEJM Image Challenge serves as a rigorous benchmark, its multiple-choice format does not replicate the full complexity of clinical practice, which typically involves unstructured data, dynamic patient interactions, and the need to generate a differential diagnosis from scratch without predefined options. Additionally, it is crucial to distinguish between high accuracy rate in standardized benchmarks and actual clinical competence. Although OpenAI o4-mini-high achieved superior accuracy, LLMs operate as predictors based on correlations, lacking the causal reasoning inherent to human cognition. Therefore, correct diagnoses in this study likely represent advanced pattern matching rather than genuine medical comprehension. This ambiguity regarding the models’ underlying processing mechanisms remains a significant limitation of current standardized benchmarks.

Third, the size of our human comparator group (*n* = 4) was inherently limited. Although the inclusion of medical students and an attending physician provided a useful preliminary benchmark, this small sample size precludes broad generalization to the wider medical community. Performance in such evaluations can be significantly influenced by individual variability in clinical exposure, medical school curricula, and test-taking strategies. In particular, the performance of a single attending physician cannot represent the average diagnostic performance of experienced clinicians. Future studies involving larger, multi-center cohorts of physicians with diverse expertise are necessary to validate these findings relative to human benchmarks.

Finally, our study relied on cases from a single, high-quality academic source. The NEJM Image Challenge consists of curated, high-quality images that do not fully represent the size, variation, and image quality (e.g., noise, artifacts, or volumetric data), characteristic of routine real-world medical imaging. The models’ high accuracy may not directly translate to real-world clinical environments, where they would face more heterogeneous, and often lower-quality data from health records and medical images.

Notwithstanding these limitations, our findings demonstrate that multimodal LLMs have achieved a high level of accuracy in standardized medical image-based benchmarks. Among the four contemporary models tested, OpenAI o4-mini-high shows strong performance, maintaining accuracy even on complex cases and with image-only inputs. The results suggest its potential to serve as a valuable adjunct in clinical practice for supporting medical education.

Future research may focus on bridging the gap between these benchmark evaluations and real-world clinical application. This includes testing models on unstructured, real-world clinical data and integrating them into clinical workflows to assess their impact on diagnostic accuracy and related patient outcomes. Furthermore, deeper investigation into the models’ output errors is also crucial. As shown in this study, most diagnostic mistakes made by OpenAI o4-mini-high were due to flawed diagnostic logic rather than fundamental processing issues. Our findings suggest that prompt engineering may be a promising avenue, as techniques like CoT and few-shot prompting corrected over half of the initial errors in our analysis. Therefore, developing strategies to mitigate these errors is a key area for future research.

In conclusion, this study demonstrates that leading multimodal LLMs achieved higher scores than human participants in this study, on a standardized medical Image Challenge. OpenAI o4-mini-high, in particular, demonstrates high performance even under image-only conditions. However, these results should be interpreted as evidence of pattern recognition capabilities rather than human-like clinical understanding. While challenges remain to be tackled before these models can be safely deployed in clinical settings, their performance in this evaluation highlights their potential for the future of clinical medicine.

## Data Availability

The raw data supporting the conclusions of this article will be made available by the authors, without undue reservation.
