# Supplementary material for: Diagnostic performance of large language models on the NEJM image challenge: a comparative study with human evaluators and the impact of prompt engineering
Source: Front Med (Lausanne). 2026 Jan 8;12:1709413. doi: 10.3389/fmed.2025.1709413 (PMC12823889; doi:10.3389/fmed.2025.1709413)
Supplement: Supplementary file 1 [file Table_1.docx]

| **Study (Ref)** | **Models Evaluated** | **Dataset / Context** | **Key Findings** |
| --- | --- | --- | --- |
| **(Ueda et al., 2024)** | GPT-4 (Text + Image input via manual description or early multimodal) | NEJM Image Challenge | Early demonstration of high accuracy; text description quality significantly impacted performance. |
| (Han et al., 2024) | GPT-4V, Gemini Pro | NEJM Image Challenge | GPT-4V demonstrated superior performance compared to text-only baselines; highlighted potential for multimodal reasoning. |
| (Kaczmarczyk et al., 2024) | GPT-4V vs. Medical Students | NEJM Image Challenge | Model outperformed students in specific radiology tasks, suggesting strong potential for education. |
| (Safavi-Naini et al., 2024) | GPT-4V, Claude 3, Llama 3, etc. | Gastroenterology Benchmarks | Comprehensive evaluation of open-source vs. proprietary models; highlighted variability across specialties. |
| (Jin et al., 2024) | GPT-4V | Multimodal Medical Benchmarks | Critical analysis revealing ‘hidden flaws’ (e.g., shallow reasoning, reliance on visual shortcuts) despite high accuracy scores. |

**Supplementary Table S1. Summary of related literature.**
