# Supplementary material for: Diagnostic performance of large language models on the NEJM image challenge: a comparative study with human evaluators and the impact of prompt engineering
Source: Front Med (Lausanne). 2026 Jan 8;12:1709413. doi: 10.3389/fmed.2025.1709413 (PMC12823889; doi:10.3389/fmed.2025.1709413)
Supplement: Supplementary file 2 [file Table_2.docx]

| Specialty | Number of Questions | Percentage (%) |
| --- | --- | --- |
| Dermatology | 30 | 15.0 |
| Infectious Diseases | 29 | 14.5 |
| Pediatrics | 19 | 9.5 |
| Clinical Immunology | 17 | 8.5 |
| Hematology | 12 | 6.0 |
| Ophthalmology | 11 | 5.5 |
| Cardiology | 11 | 5.5 |
| Pulmonology | 10 | 5.0 |
| Neurology | 8 | 4.0 |
| Oncology | 8 | 4.0 |
| Nephrology | 7 | 3.5 |
| Gastroenterology | 5 | 2.5 |
| General Surgery | 5 | 2.5 |
| General Internal Medicine | 5 | 2.5 |
| Endocrinology | 5 | 2.5 |
| Vascular Surgery | 4 | 2.0 |
| Thoracic Surgery | 4 | 2.0 |
| Orthopedics | 4 | 2.0 |
| Dentistry | 3 | 1.5 |
| Obstetrics and Gynecology | 2 | 1.0 |
| Otorhinolaryngology (ENT) | 1 | 0.5 |

**Supplementary Table S2. Distribution of questions by medical specialty (n=200)**
