# Supplementary material for: Diagnostic performance of large language models on the NEJM image challenge: a comparative study with human evaluators and the impact of prompt engineering
Source: Front Med (Lausanne). 2026 Jan 8;12:1709413. doi: 10.3389/fmed.2025.1709413 (PMC12823889; doi:10.3389/fmed.2025.1709413)
Supplement: Supplementary file 3 [file Table_3.docx]

| Question number / Release date | Provided response | Error type |
| --- | --- | --- |
| Q18 / 2025.1.30 | The most likely diagnosis is **Geographic tongue**.  **Rationale:** The image shows irregular, smooth, depapillated (pink) “map-like” patches on the dorsal tongue within a background that can appear mildly whitish—classic for benign migratory glossitis (geographic tongue). These lesions often shift over time and can cause discomfort or burning. They are not wiped away because they represent loss of filiform papillae, not a removable coating.  The history of **painful scrotal ulcers** that have resolved argues against primary syphilis (which classically is **painless**) and makes secondary syphilis less likely as the cause of the tongue findings—secondary syphilis oral mucous patches tend to be grayish, translucent, and often accompanied by systemic rash or other stigmata, which are not described here. Other choices: oral leukoplakia is a persistent white plaque (not erythematous map-like areas), lichen planus has a lacy reticular pattern, and squamous cell carcinoma would be more focal and not migratory in appearance. | Diagnostic logic / Decision-making output |
| Q28 / 2024.11.21 | Most likely diagnosis: **Irritant contact dermatitis.**  **Reasoning:** This is an acute, painful, burning erythematous reaction localized to the dorsal thumbs and adjacent fingers with a focal blister—consistent with a direct chemical or physical irritant exposure. The symmetrical/paired involvement of both hands (common when someone uses both hands to handle a substance) and the confluent erythema (rather than the more streaky or bizarre patterns seen in phytophotodermatitis) favors an acute irritant injury. Phytophotodermatitis usually follows plant sap exposure plus sun and often produces irregular pigmentation or streaks; porphyria cutanea tarda is more chronic with skin fragility and bullae on sun-exposed areas, not a sudden 2-day burning rash. Atopic dermatitis would be more chronic and eczematous, and hand-foot-and-mouth disease would have vesicles in characteristic locations and systemic prodrome, which are lacking here. | Diagnostic logic / Decision-making output |
| Q41 / 2024.8.22 | Based on the clinical context and most consistent ECG pattern, this is **posterior myocardial infarction**.  **Reasoning:** Posterior MI often presents with **horizontal ST depressions in V1–V3** which are actually reciprocal changes to ST elevation “behind” the standard precordial leads. There may also be tall R waves in those leads (mirror of Q waves) and upright T’s. The relatively acute presentation (1 hour of chest pain), tachycardia, and even the mild troponin rise are compatible with early evolving infarction. Confirmation would come from placing posterior leads (V7–V9), which would show ST elevation. | Diagnostic logic / Decision-making output |
| Q61 / 2024.4.4 | Answer: Rifampin.  Rifampin is known to cause orange-red discoloration of body fluids — but less commonly, in darker tissues like oral mucosa, this can appear brownish or bluish, especially in certain lighting or with concurrent iron deposition or melanin changes.There are case reports of rifampin-induced oral mucosal pigmentation appearing after several weeks. The CNS symptoms (anxiety, insomnia, tremor) are also known but rare side effects of rifampin, related to its effect on the nervous system and possible drug–drug interactions. | Diagnostic logic / Decision-making output |
| Q70 / 2024.2.1 | I’m going to need a bit more context to pick the most appropriate next test—could you describe the clinical presentation (including relevant history and exam findings) and summarize what the image shows? | Input processing / Multimodal alignment |
| Q111 / 2023.4.20 | The radiographs show a both-bone forearm fracture (radius and ulna shaft) impairing pronation/supination—classic for a high-energy injury such as a fall on an outstretched hand. Answer: Fall.  Brief rationale:  Simultaneous diaphyseal fractures of both radius and ulna in an adult usually reflect a direct or indirect traumatic event (e.g., fall), not low-energy osteoporotic fragility patterns (which more often involve distal radius or vertebrae) or repetitive strain.  There is no radiographic evidence of a lytic/blastic lesion to suggest malignancy causing a pathologic fracture.  Assault (e.g., “nightstick” injury) typically produces an isolated ulnar shaft fracture, not both bones. | Diagnostic logic / Decision-making output |
| Q123 / 2023.1.26 | Most likely diagnosis: Arachnoid cyst  Brief explanation: The MRI shows a large, well-demarcated, extra-axial, CSF-intensity lesion causing mass effect with remodeling of adjacent bone—consistent with an arachnoid cyst. Cystic echinococcosis and neurocysticercosis are typically intra-parenchymal with daughter cysts or scolex features; a colloid cyst localizes to the foramen of Monro, and a pineal cyst is midline in the pineal region. | Diagnostic logic / Decision-making output |
| Q125 / 2023.1.12 | Most likely etiology: Ileocolic intussusception  Reasoning: The CT images show the classic “target”/ “sausage” appearance with telescoping of bowel loops consistent with intussusception. In a 5-year-old with intermittent abdominal pain and otherwise benign exam, the most common pediatric pattern involving the terminal ileum prolapsing into the colon is ileocolic intussusception. | Diagnostic logic / Decision-making output |
| Q143 / 2022.9.8 | Most likely diagnosis: Pericardial cyst.  Brief rationale: A well-circumscribed, slowly enlarging, asymptomatic mass along the left heart border on serial chest radiographs is classic for a pericardial cyst. These are benign congenital or acquired fluid-filled lesions that can remain stable or grow gradually. Other choices (e.g., mediastinal hematoma or thrombus) would be expected to present more acutely or with different anatomic relationships, and a teratoma would more often show heterogeneous density. | Diagnostic logic / Decision-making output |
| Q153 / 2022.6.30 | Palla sign.  Explanation: The image shows enlargement of the right descending pulmonary artery (“sausage-shaped”), which is classically described as Palla sign and can be seen in acute pulmonary embolism. Given her history of pulmonary embolism risk factors (ulcerative colitis, prior PE) and acute pleuritic chest pain with cough, this fits an acute embolic event. | Input processing / Multimodal alignment. |
| Q167 / 2022.3.24 | Diagnosis: Livedo racemosa.  Rationale: The persistent, irregular, broken netlike mottling that doesn’t vary with temperature fits livedo racemosa (as opposed to physiologic livedo reticularis). The biopsy shows intravascular tumor emboli (CK7+, GATA3+ consistent with breast carcinoma) causing vascular occlusion and the livedo-pattern—secondary livedo racemosa from cutaneous intravascular metastasis. | Diagnostic logic / Decision-making output |
| Q184 / 2021.11.25 | Most likely etiology: Rhinolith  Brief rationale: A hard, white, radiodense mass in the nasal cavity with chronic unilateral obstruction is classic for a rhinolith—mineral deposition around a nidus (e.g., foreign body or dried secretions). | Diagnostic logic / Decision-making output |

**Supplementary Table S3. Question IDs, model-provided responses, and assigned error types for the 12 incorrect answers generated by OpenAI o4-mini-high.**
