# Supplementary figures and images for: Diagnostic performance of large language models on the NEJM image challenge: a comparative study with human evaluators and the impact of prompt engineering
Source: Front Med (Lausanne). 2026 Jan 8;12:1709413. doi: 10.3389/fmed.2025.1709413 (PMC12823889; doi:10.3389/fmed.2025.1709413)

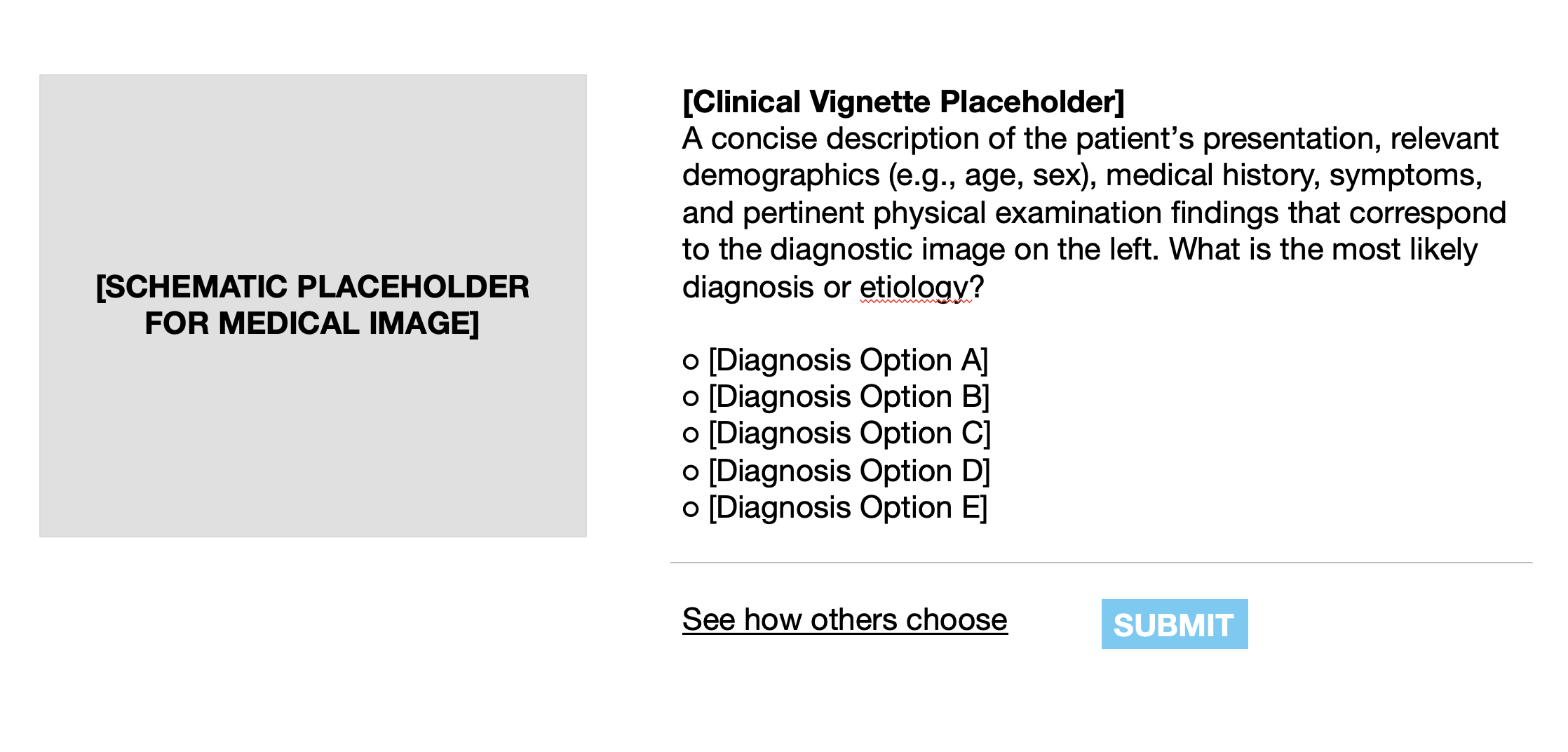

Supplement: Supplementary file 4 [file Image_1.png]
