# Supplementary material for: Diagnostic performance of large language models on the NEJM image challenge: a comparative study with human evaluators and the impact of prompt engineering
Source: Front Med (Lausanne). 2026 Jan 8;12:1709413. doi: 10.3389/fmed.2025.1709413 (PMC12823889; doi:10.3389/fmed.2025.1709413)
Supplement: Supplementary file 5 [file Supplementary_file_1.docx]

**CoT prompt：**

Act as an experienced physician and professor at a prestigious university hospital. Your task is to answer questions based on medical images and patient case descriptions. Follow this structure and explicitly show your chain of thought:

1. Observation

– Describe in detail the key findings on the image (e.g. “I see a left pleural effusion with blunting of the costo-phrenic angle…”)

– Summarize the salient points of the clinical vignette (age, gender, symptoms, labs, history).

2. Hypothesis Generation

– Based on these observations, list all plausible diagnoses or answer choices.

– For each, note why it could explain the findings.

3. Evidence Weighing

– For each hypothesis, review supporting and contradictory clues from the image and case.

– Reference specific features (e.g. “The absence of vascular redistribution makes cardiogenic edema less likely.”).

4. Differential Narrowing

– Compare the strength of evidence across hypotheses.

– Eliminate less likely options one by one, stating your rationale.

5. Conclusion

– State the most likely diagnosis or the answer choice you select.

– Give a one-sentence summary linking back to the key distinguishing feature.

Output format:

Thought 1 (Observation): …

Thought 2 (Hypotheses): …

Thought 3 (Weighing): …

Thought 4 (Narrowing): …

Final Answer: …

Now, here is the question. Please think step by step and show your full reasoning before giving the final answer.

**Q18 2025.1.30**

Thought 1 (Observation):
– On the dorsal surface of the tongue I see multiple discrete, smooth, pink-to-erythematous macules (“guttate” spots) embedded in a more diffuse gray-whitish pseudomembranous coating. These spots are sharply demarcated and do not wipe off with scraping.
– Clinically, this is a 29-year-old man with well-controlled HIV who developed a 1-week history of painful whitish tongue changes, preceded 4 weeks ago by painful scrotal ulcers that have since resolved. No active genital lesions or lymphadenopathy are found today.

Thought 2 (Hypotheses):
– **Geographic tongue (benign migratory glossitis):** Erythematous, map-like areas of papillary atrophy with white borders on the tongue.
– **Lichen planus:** Reticular (“lace-like”) white striae or papules on the buccal mucosa or tongue, often non-erosive.
– **Oral leukoplakia:** Homogenous white plaques on the oral mucosa that cannot be scraped off and are potentially premalignant.
– **Secondary syphilis (mucous patches):** Gray-white, oval or serpiginous mucosal patches/macules that appear weeks to months after a primary chancre.
– **Squamous cell carcinoma:** Usually presents as a persistent ulcer or mass, often on the lateral tongue, not multiple symmetric macules.

Thought 3 (Weighing):
– **Geographic tongue** can cause smooth erythematous patches with white borders, but these are typically asymptomatic or mildly discomforting, migratory, and lack an associated systemic prodrome.
– **Lichen planus** shows a reticular, network-like pattern (Wickham striae), not discrete pink macules embedded in a coating.
– **Oral leukoplakia** presents as uniform white plaques without underlying pink macules, and is usually painless.
– **Secondary syphilis** classically causes “mucous patches”—grey-white, painless or mildly painful plaques/macules on oral mucosa appearing 4–10 weeks after the primary chancre. The prior history of painful genital ulcers 4 weeks ago fits a primary chancre, and the timing corresponds perfectly to the onset of secondary lesions.
– **Squamous cell carcinoma** would present as a persistent solitary ulcer or mass and is very unlikely to produce multiple discrete lesions.

Thought 4 (Narrowing):
– Geographic tongue and lichen planus lack the timeline and systemic context of a prior chancre.
– Oral leukoplakia’s appearance and natural history don’t match the acute, painful “guttate” changes.
– Squamous cell carcinoma’s morphology (solitary mass) and epidemiology (older patients, risk factors) don’t fit.
– Secondary syphilis alone explains the timing (4 weeks post-chancre), the morphology of mucous patches, and the systemic course in an HIV-infected patient.

Final Answer: Secondary syphilis. The discrete pink macules within a gray-white coating are mucous patches of secondary syphilis appearing weeks after a primary chancre.

**Q28 2024.11.21**

Thought 1 (Observation):
– The images show bilateral involvement of the dorsum of the hands, most prominently over the thumbs and extending in a confluent erythematous band to the medial aspect of the second fingers, with more scattered erythematous macules over the knuckles and adjacent digits. There is a single tense, clear blister at the base of the left thumb.
– Clinically, this is a 40-year-old man with a 2-day history of a burning (rather than intensely pruritic) rash, limited to both hands, with no mention of oral lesions, foot lesions, or chronic skin disease.

Thought 2 (Hypotheses):
– **Atopic dermatitis**: typically chronic, intensely pruritic, with a personal/family history of atopy, and favors flexural surfaces rather than an acute dorsal hand eruption.
– **Hand, foot, and mouth disease**: viral exanthem in children/adolescents with vesicles on hands, feet, and oral mucosa; this adult has no oral or foot lesions.
– **Irritant contact dermatitis**: acute burning erythema and occasional blistering at sites of direct irritant exposure; distribution corresponds to areas most likely in contact with the offending agent.
– **Phytophotodermatitis**: phototoxic reaction after contact with certain plant compounds plus UV exposure, causing erythema, blistering, often with irregular streaks and subsequent hyperpigmentation; requires history of plant contact and sun exposure.
– **Porphyria cutanea tarda**: chronic blistering/photosensitivity of sun-exposed skin (especially dorsal hands), often with skin fragility, hyperpigmentation, hypertrichosis, and abnormal porphyrin labs.

Thought 3 (Weighing):
– **Atopic**: lack of chronicity or pruritus, and distribution on dorsal hands is atypical.
– **HFMD**: absence of oral lesions and foot involvement argues strongly against it.
– **Irritant CD**: acute burning, sharp distribution matching contact sites, blister formation all support this.
– **Photophoto**: no history of plant exposure or sun‐linked timing; lesions are fairly uniform bands rather than irregular photodistributed streaks.
– **PCT**: lesions here are acute and lack the classic chronic skin fragility, hyperpigmentation, and hypertrichosis; no lab data or chronic pattern.

Thought 4 (Narrowing):
– Exclude atopic dermatitis for lack of chronic/itchy history.
– Exclude HFMD given adult age and absent mucocutaneous findings.
– Exclude phytophotodermatitis and PCT based on missing trigger history (sun/plant) and chronic features.

Final Answer: Irritant contact dermatitis — the acute burning erythema and localized blistering in a pattern matching direct exposure to an irritant on the dorsal thumbs and adjacent fingers is most consistent with an irritant contact reaction.

**Q41 2024.8.22**

Thought 1 (Observation):
– The ECG shows diffuse ST-segment elevations with a concave (“saddle-shaped”) morphology in multiple leads—limb leads I, II, III, aVL, aVF and precordials V2–V6—without a clear regional pattern. There are PR-segment depressions in several leads (especially II, aVL, V4–V6) and reciprocal PR-elevation/ST-depression in aVR. There are no localized Q-waves or marked reciprocal ST elevations.
– Clinical vignette: 57-year-old man, 1 hour of chest pain, heart rate 125, blood pressure 128/84, O_2_ sat 99% on room air, normal exam, minimally elevated troponin I (35 ng/L; ULN 34).

Thought 2 (Hypotheses):

1. **Anteroseptal STEMI** – Would produce ST elevation localized to V1–V4 (territorial).
2. **Diffuse subendocardial ischemia** – Typically manifests as widespread horizontal ST depressions with ST elevation in aVR.
3. **Pericarditis** – Classically causes diffuse concave ST-elevations + PR depressions across most leads, reciprocal changes in aVR.
4. **Posterior myocardial infarction** – Shows ST depressions and tall R waves in V1–V3 (reciprocal), with ST elevation in posterior leads (not seen here).
5. **Wellens syndrome** – Characterized by biphasic or deeply inverted T-waves in V2–V3, minimal ST changes, pain-free.

Thought 3 (Weighing):

- **Anteroseptal STEMI:** ST elevations are not confined to V1–V4; here they are diffuse. No localized Q‐waves or reciprocal inferior depressions.
- **Diffuse subendocardial ischemia:** We do not see diffuse ST depressions; instead we see elevations.
- **Pericarditis:** Diffuse concave ST elevations + PR depressions are textbook. Reciprocal PR/ST changes in aVR support this.
- **Posterior MI:** We would expect horizontal ST depressions in V1–V3 only, not elevations in V2–V6.
- **Wellens syndrome:** There are no biphasic or deeply inverted T-waves; moreover, there is frank ST elevation.

Thought 4 (Narrowing):
– The only pattern matching diffuse, concave ST elevations with PR depressions across multiple territories (and reciprocal changes in aVR) is pericarditis. All other diagnoses either localize to specific leads or show predominantly ST depressions/inverted T-waves.

Final Answer: Pericarditis—demonstrated by the diffuse concave ST-segment elevations with PR-segment depressions and reciprocal aVR changes.

**Q61 2024.4.4**

Thought 1 (Observation):
– A 35-year-old man started multidrug therapy for leprosy one month ago and now, over the past 4 days, has developed anxiety, insomnia, and a resting tremor of his hands and feet.
– On exam he has bluish (cyanotic) discoloration of the lips and tongue, consistent with impaired oxygen-carrying capacity rather than a purely dermatologic pigment change.

Thought 2 (Hypotheses):

1. **Dapsone** – Known to cause oxidative stress on hemoglobin leading to methemoglobinemia and cyanosis.
2. **Clofazimine** – Causes reddish-brown to black pigment deposition in skin and mucosa, but not true cyanosis; no prominent neuroexcitation features.
3. **Rifampin** – Causes orange-red discoloration of secretions, not blue; neuro side effects are uncommon.
4. **Thalidomide** – Can cause peripheral neuropathy over months, sedation, but not cyanosis.
5. **Salicylates** – Toxicity produces tinnitus, hyperventilation, metabolic acidosis, but not bluish mucosal cyanosis.

Thought 3 (Weighing):

- **Dapsone**: Oxidizes hemoglobin to methemoglobin → reduced O₂ delivery → cyanosis, central hypoxia can manifest as anxiety, restlessness, tremor.
- **Clofazimine**: Pigment deposits produce brownish discoloration of skin, not a true blue cyanosis; no associated tremor/insomnia.
- **Rifampin**: Leaves orange urine, tears, saliva; lip/tongue would appear orange, not blue.
- **Thalidomide**: Neuropathy is sensory, insidious; no mucosal color change.
- **Salicylates**: No mucosal pigmentation; primary neuro signs are hyperpnea, tinnitus, confusion rather than isolated tremor and insomnia.

Thought 4 (Narrowing):
– Only dapsone both oxidizes hemoglobin to cause true cyanosis of the lips/tongue and can produce symptoms of central hypoxia (anxiety, tremor, insomnia). All other agents cause different pigment changes or neurotoxicities that don’t match this picture.

Final Answer: Dapsone. This drug’s propensity to induce methemoglobinemia explains the bluish mucosal cyanosis and hypoxia-driven neuro symptoms.

**Q70 2024.2.1**

Thought 1 (Observation):
– A 44-year-old man developed 3 days of vision loss and ocular pain after 3 hours of direct pressure on his left eye (prolonged unconsciousness).
– On exam: hemorrhagic chemosis, fixed mid-dilated pupil, normal intraocular pressure; fundus shows diffuse retinal whitening (full-thickness edema), MRI shows engorged orbital tissues and extraocular muscles = orbital compartment syndrome with retinal and choroidal ischemia.

Thought 2 (Hypotheses):

1. **Absence of light perception** – with complete retinal/choroidal infarction, no visual perception remains.
2. **Relative afferent pupillary defect (RAPD)** – an afferent pathway lesion (retina/optic nerve) yields an RAPD.
3. **Complete ophthalmoplegia** – orbital compartment syndrome compresses cranial nerves III/IV/VI or muscles → paralysis of extraocular movements.
4. **Proptosis** – increased intra-orbital pressure and tissue engorgement should push the globe forward.
5. **Vertical nystagmus** – a disconjugate rhythmic oscillation, usually of central (brainstem/cerebellar) origin.

Thought 3 (Weighing):
– **No light perception**: retinal infarction abolishes vision entirely → strongly expected.
– **RAPD**: loss of afferent input from the ischemic retina produces a Marcus Gunn pupil → expected.
– **Ophthalmoplegia**: compartment syndrome compresses the nerves/muscles → eye cannot move → expected.
– **Proptosis**: engorged orbital contents → anterior displacement → expected.
– **Vertical nystagmus**: requires a central vestibular/cerebellar lesion, not isolated orbital compression → not explained here.

Thought 4 (Narrowing):
All listed findings (no light perception, RAPD, ophthalmoplegia, proptosis) stem directly from orbital compartment syndrome and retinal/choroidal ischemia. Vertical nystagmus does not arise from an orbital process.

Final Answer: Vertical nystagmus is least likely to be found.

**Q111 2023.4.20**

Thought 1 (Observation):
– The AP and lateral radiographs show a single, transverse, non-displaced fracture of the ulnar diaphysis with intact overlying skin and preserved alignment of the radius and distal radioulnar joint.
– Clinically, the patient has isolated forearm pain, swelling, and pain exacerbated by passive pronation–supination—findings localizing the injury to the forearm shafts rather than the wrist or elbow.

Thought 2 (Hypothesis Generation):
Possible contributory factors for an isolated ulnar shaft fracture include:

1. **Direct blow** (e.g., defensive “nightstick” fracture in assault)
2. **Fall on an outstretched hand (FOOSH)**
3. **Pathologic fracture from malignancy**
4. **Fragility fracture from osteoporosis**
5. **Stress (repetitive) fracture**

Thought 3 (Evidence Weighing):

- **Direct blow/Assault:** Classic nightstick fractures result from a direct strike to the ulna, producing an isolated ulnar shaft break with minimal displacement and no joint involvement—exactly what we see.
- **FOOSH:** Typically causes distal‐radius (“Colles”) fractures or combined radius/ulna injuries, not an isolated mid-shaft ulna.
- **Malignancy:** Pathologic fractures often show cortical thinning, lytic or blastic lesions; here the cortex is otherwise normal.
- **Osteoporosis:** Predisposes to metaphyseal fragility fractures (wrist, hip, spine), not clean mid-shaft diaphyseal breaks.
- **Repetitive strain:** Ulnar stress fractures are very rare and occur in specific athletes (e.g. bowlers); they present as hairline cracks, not a full transverse break.

Thought 4 (Differential Narrowing):
– The radiographic pattern—isolated, transverse ulnar mid-shaft fracture with an otherwise normal-appearing bone—is pathognomonic for a direct blow (nightstick) injury. All other mechanisms produce different fracture patterns or radiographic bone changes.

Final Answer: Assault (direct blow to the ulna) is the most likely contributory factor.

**Q123 2023.1.26**

Thought 1 (Observation):
– On MRI there is a very large, well‐circumscribed cystic lesion with CSF‐like signal (hypointense on T1, hyperintense on T2) that is multilocular – you can clearly see smaller “daughter” cysts within a larger mother cyst. There is mass effect on adjacent brain but no significant surrounding edema.
– Clinically, a 14-year-old boy from a farm has 1 month of episodic headaches and vomiting, signs of slowly progressive intracranial hypertension.

Thought 2 (Hypotheses):

1. **Arachnoid cyst** – congenital, extra-axial CSF cyst, usually unilocular, no internal septations.
2. **Colloid cyst** – small, at the foramen of Monro, not multilocular or this large.
3. **Cystic echinococcosis (hydatid cyst)** – can form large spherical cysts with daughter cysts in brain; risk increased in farm dwellers.
4. **Neurocysticercosis** – multiple small parenchymal cysts (“rice-grain”), often with scolex; rarely a giant solitary multilocular cyst.
5. **Pineal cyst** – small, benign, in pineal region; does not form huge multiloculated masses.

Thought 3 (Weighing):

- **Arachnoid cyst:** lacks daughter cysts/rosette appearance.
- **Colloid/pineal cyst:** far too small and in wrong location.
- **Neurocysticercosis:** typically many small cysts, sometimes calcified, not one giant multilocular.
- **Cystic echinococcosis:** classic large mother cyst with internal daughter cysts on T1/T2, matches farm exposure.

Thought 4 (Narrowing):
All other possibilities fail to explain the multilocular “daughter” cyst morphology or the farm‐associated exposure. The imaging and epidemiology are pathognomonic for hydatid disease.

Final Answer: Cystic echinococcosis.

**Q125 2023.1.12**

Thought 1 (Observation):
– The axial CT image shows a classic “target” or “doughnut” sign in the mid-abdomen, with concentric rings of bowel wall. The sagittal image reveals a tubular, “sausage-shaped” mass of telescoped bowel.
– Clinically, a previously healthy 5-year-old boy has 2 days of intermittent periumbilical pain without peritoneal signs, and the initial ultrasound was reportedly normal.

Thought 2 (Hypotheses):

1. **Crohn’s disease** – would show segmental bowel wall thickening, skip areas, fat creeping, not a target sign.
2. **Colocolonic intussusception** – intussusception confined to colon, less common in this age group.
3. **Colonic pseudo-obstruction** – massive colonic dilation with transition zones, no concentric rings.
4. **Ileocolic intussusception** – most common pediatric intussusception; produces the “target” and “sausage” signs.
5. **Sigmoid volvulus** – shows a “coffee-bean” sign and whirl of mesenteric vessels in sigmoid, rare in young children.

Thought 3 (Weighing):

- **Crohn’s disease:** lacks abrupt telescoping and characteristic target/sausage morphology.
- **Colocolonic intussusception:** technically similar imaging but far less common in 5-year-olds, and usually involves more distal colon.
- **Colonic pseudo-obstruction:** would demonstrate massive distention of colon without focused mass.
- **Ileocolic intussusception:** textbook appearance of concentric rings on axial and a sausage on sagittal CT, fits the age and intermittent pain.
- **Sigmoid volvulus:** would show a bent inner tube/coffee-bean in pelvis, not a target lesion in the mid-abdomen.

Thought 4 (Narrowing):
– The imaging findings—doughnut on axial and sausage on sagittal—are pathognomonic for intussusception, and in a 5-year-old the ileocolic form is by far the predominant type.

Final Answer: Ileocolic intussusception.

**Q143 2022.9.8**

Thought 1 (Observation):
– On the PA chest radiograph there is a well-circumscribed, round mass abutting the left heart border at the cardiophrenic angle. Over serial films spanning 6 years it has grown slowly but remains smooth and non-calcified, with no evidence of invasive margins or bone destruction.
– The patient is a 79-year-old woman, asymptomatic, with this finding discovered incidentally and monitored without any signs of systemic illness, trauma, or acute change.

Thought 2 (Hypotheses):

1. **Giant coronary aneurysm** – a saccular dilatation of a coronary artery could bulge against the cardiac silhouette.
2. **Left atrial appendage thrombus** – a clot in the atrial appendage might project near the left heart border.
3. **Mediastinal hematoma** – accumulation of blood in the mediastinum, typically post-trauma or rupture.
4. **Pericardial cyst** – a benign congenital fluid-filled cyst in the pericardial recess, often at a cardiophrenic angle.
5. **Teratoma** – an anterior mediastinal germ cell tumor that may show mixed density and slow growth.

Thought 3 (Weighing):

- **Giant coronary aneurysm:** These are rare, often related to atherosclerosis or vasculitis (e.g., Kawasaki), but they usually have irregular borders or calcifications and would be associated with anginal symptoms or ischemia—none of which this patient has.
- **Left atrial appendage thrombus:** Cannot be reliably seen on chest X-ray; diagnosis is made by echocardiography. Also, a thrombus wouldn’t progressively enlarge over years without causing embolic events (she had a prior stroke but that predates this 6-year stability).
- **Mediastinal hematoma:** Presents acutely with pain or hemodynamic compromise after trauma or vessel rupture; it would resolve or evolve over weeks rather than persist and grow slowly for years.
- **Pericardial cyst:** Classically appears as a sharply marginated, water-density lesion at a cardiophrenic angle, is congenital, asymptomatic, and can enlarge slowly.
- **Teratoma:** Often shows fat, calcification, or soft-tissue elements on imaging; typically occurs in younger patients and may produce mass effect on adjacent structures.

Thought 4 (Narrowing):
Only a pericardial cyst explains a smooth, non-calcified, slowly enlarging, asymptomatic mass at the left cardiophrenic angle over many years without acute symptoms or systemic signs. All other entities either present acutely, have different imaging characteristics, or occur in different patient populations.

Final Answer: Pericardial cyst.

**Q153 2022.6.30**

Thought 1 (Observation):
– On the PA chest radiograph there is a peripheral, wedge-shaped, pleural-based opacity in the left lower lung field, with its base abutting the pleura and its apex pointing toward the hilum.
– The patient is a young woman with risk factors for thromboembolism (history of pulmonary embolism, ulcerative colitis) presenting with pleuritic chest pain and dry cough.

Thought 2 (Hypotheses):
Possible named radiographic signs of pulmonary embolism include:

1. **Hampton’s hump** – a peripheral, wedge-shaped opacity representing pulmonary infarction.
2. **Westermark sign** – focal oligemia (increased lucency) distal to a pulmonary arterial occlusion.
3. **Fleischner sign** – enlargement of the central pulmonary artery due to pressure from an embolus.
4. **Palla sign** – enlargement of the right descending pulmonary artery on a frontal radiograph.
5. **Spine sign** – increased density of the lower thoracic vertebrae on a lateral film, indicating pleural fluid.

Thought 3 (Weighing):
– The lesion here is an opacity, not an area of increased lucency, so it cannot be Westermark’s sign.
– There is no obvious enlargement of the main or right descending pulmonary artery, so Fleischner and Palla signs are not exemplified.
– The image is a frontal view, so the lateral-film spine sign does not apply.
– A peripheral, wedge-shaped opacity exactly matches the description of Hampton’s hump.

Thought 4 (Narrowing):
– Only Hampton’s hump describes a pleural-based, wedge-shaped infarct opacity on a PA chest radiograph in the setting of pulmonary embolism.

Final Answer: Hampton’s hump.

**Q167 2022.3.24**

Thought 1 (Observation):
– The patient has a purplish, reticulated, net-like discoloration of the facial skin following cholesterol embolization. The pattern is persistent and irregular rather than a uniform mottling from cold exposure.

Thought 2 (Hypotheses):
– **Cutis marmorata**: transient physiologic mottling in response to cold, resolves with warming.
– **Harlequin syndrome**: unilateral flushing and sweating of the face/trunk due to autonomic dysfunction, not a reticular pattern.
– **Telangiectasia**: focal dilated capillaries (“spider veins”), not a broad net-like pattern.
– **Livedo reticularis**: a mottled, net-like cyanotic pattern, often benign and transient.
– **Livedo racemosa**: a pathologic, irregular, branching reticular pattern that is persistent and associated with vaso-occlusive disorders.

Thought 3 (Weighing):
– The pattern here is persistent, asymmetric, and associated with cholesterol emboli (a vaso-occlusive process).
– Cutis marmorata and livedo reticularis tend to be more uniform and reversible; telangiectasias are small and focal; Harlequin is hemispheric flushing.
– Livedo racemosa is the pathological reticular rash seen in cholesterol embolization.

Thought 4 (Narrowing):
– Only livedo racemosa matches a persistent, irregular net-like cyanosis in the setting of cholesterol microembolization.

Final Answer: Livedo racemosa.

**Q184 2021.11.25**

Thought 1 (Observation):
– On rhinoscopy there is a hard, nontender white mass lodged on the floor of the right nasal cavity.
– CT shows a well-defined radiodense lesion lying on the nasal floor, separate from the bony septum and maxilla, without evidence of a tooth socket.

Thought 2 (Hypotheses):

1. **Calcified polyp** – would be soft-tissue based, not uniformly radiodense.
2. **Enchondroma** – cartilaginous tumor, usually shows chondroid (“rings and arcs”) calcification, not a solid opaque mass.
3. **Osteoma** – arises from sinus walls (commonly frontal), not on the nasal floor, and would be contiguous with cortical bone.
4. **Rhinolith** – calcified concretion formed around a nidus in the nasal cavity, characteristically lies on the nasal floor and is fully radiopaque.
5. **Ectopic tooth** – appears as a tooth-shaped opacity, often with identifiable crown/root morphology within the maxillary sinus or palate.

Thought 3 (Weighing):
– The lesion is freely lying on the nasal floor (not attached to periosteum), uniformly radiodense, and the patient has septal spurs/perforation likely from chronic pressure.
– No tooth morphology or sinus origin to suggest an osteoma or ectopic tooth.
– The appearance and location exactly fit a rhinolith.

Thought 4 (Narrowing):
– Only a rhinolith explains a freely mobile, uniformly calcified mass on the floor of the nasal cavity formed around a long-standing nidus.

Final Answer: Rhinolith.

**Few-shot prompt:**

Act as an experienced physician and professor at a prestigious university hospital. Your task is to answer questions based on medical images and patient case descriptions. Utilize your expertise to accurately interpret the information and provide the most likely diagnosis or answer choice. Use the following examples as guides. (Note: Three representative cases from the NEJM Image Challenge published on May 29, May 22, and May 15, 2025 were used as few-shot demonstrations. The model was provided with the full case details, including images and official explanations. Due to copyright restrictions, the original content of these cases is not reproduced here. Readers may refer to the official website for details.)

**Q18 2025.1.30**

The patient’s history of a genital chancre about a month ago followed by the appearance of multiple flat, non-wipable white plaques studded with smooth, pink “guttate” macules on the dorsal tongue is classic for mucous patches of secondary syphilis.

**Q28 2024.11.21**

The sharply demarcated, burning erythema with a single focal blister localized to the exact area of hand contact—and sparing of non‐contact sites—is most consistent with an acute irritant contact dermatitis rather than a generalized photosensitivity (porphyria), viral exanthem (HFMD), or chronic dermatosis (atopic dermatitis, PCT). Phytophotodermatitis tends to produce bizarre, streaky lesions after plant-Juice + sun exposure, and PCT produces chronic fragility with milia and scarring. This pattern—especially the web-space/thumb region where an irritant (chemical or glove lubricant, for example) would touch—is classic for irritant contact dermatitis.

**Q41 2024.8.22**

The tracing shows widespread horizontal ST‐segment depressions in the limb leads and precordial leads with reciprocal ST‐segment elevation in aVR rather than localized ST elevation in V₁–V₄ (which you’d see in an anteroseptal STEMI) or the discrete ST depressions in V₁–V₃ with tall R-waves of a posterior infarct. This diffuse downsloping ST depression pattern with ST elevation in aVR is classic for subendocardial (non–transmural) ischemia—most often from critical left main or severe multivessel disease.

**Q61 2024.4.4**

The striking cyanosis of the lips and tongue in the absence of cardiopulmonary disease—along with new-onset anxiety, insomnia, and a fine resting tremor—is most consistent with methemoglobinemia due to dapsone. Dapsone’s oxidative metabolites convert hemoglobin to methemoglobin, which cannot bind O₂, producing a “chocolate-blue” mucosal discoloration and symptoms from tissue hypoxia. None of the other agents used in leprosy therapy cause this picture.

**Q70 2024.2.1**

Vertical nystagmus is the least likely finding.

In this case of orbital compression with resulting ischemic injury, you would expect:

- **No light perception** from full‐thickness retinal infarction.
- **A relative afferent pupillary defect** (afferent pathway loss).
- **Complete ophthalmoplegia** from extraocular muscle and/or cranial nerve ischemia.
- **Proptosis** from orbital tissue edema and venous congestion.

Vertical nystagmus, however, is a central (brainstem or cerebellar) sign and would not arise from an isolated orbital/retinal ischemic injury.

**Q111 2023.4.20**

The sharply transverse, non‐comminuted midshaft fracture of the radius—without evidence of periosteal reaction, lytic lesion, or cortical thinning—is most characteristic of a single, direct‐blow injury (bending force) rather than a fall onto an outstretched hand (which tends to produce oblique or comminuted patterns at the distal radius), a pathologic fracture from malignancy (which would show an underlying lesion), osteoporosis (which more commonly causes distal radius “Colles” fractures), or a stress fracture (which shows periosteal reaction). This pattern is classically seen with assault (a direct blow to the forearm).

**Q123 2023.1.26**

The “snow-man” appearance of a large, well-demarcated CSF-signal cyst containing multiple daughter cysts in a patient with farm exposure is classic for a hydatid cyst (cystic echinococcosis). Neurocysticercosis produces multiple small cysts (often with visible scoleces) rather than a single giant multiloculated cyst. Arachnoid, pineal, or colloid cysts are unilocular, and none reach this size or contain daughter cysts.

**Q125 2023.1.12**

The concentric “target” lesion on the axial image (Panel A) and the tubular “sausage” mass on the sagittal reconstruction (Panel B) are classic CT signs of an intussusception. In a 5-year-old with episodic periumbilical pain and an otherwise normal workup, the most common form is ileocolic intussusception, with the terminal ileum telescoping into the colon.

**Q143 2022.9.8**

A slowly enlarging, sharply demarcated, water-density mass at the left cardiophrenic angle over many years is virtually pathognomonic for a pericardial cyst. None of the other options (coronary aneurysm, atrial thrombus, hematoma, or teratoma) produce a well-defined, benign-appearing, fluid-density lesion that grows so indolently along the cardiac border.

**Q153 2022.6.30**

The chest radiograph shows a focal area of decreased vascularity (oligemia) in the right lung field without other parenchymal abnormality, which is the classic **Westermark sign** of pulmonary embolism.

**Q167 2022.3.24**

The mottled, violaceous, branching (“net‐like”) but irregular and persistent pattern of skin discoloration here is characteristic of **livedo racemosa**, a pathologic form of livedo often seen with cholesterol embolization.

**Q184 2021.11.25**

The lesion’s uniform, enamel‐level radiodensity and characteristic crown‐and‐root morphology on CT (white arrow) in the nasal floor are diagnostic of an ectopic intranasal tooth. None of the other entities (rhinolith, osteoma, calcified polyp, enchondroma) show that classic tooth structure.
